# Domain C: Systems of Economic Exchange

**DOI:** 10.1007/978-3-030-61315-0_6

**Published:** 2020-09-22

**Authors:** Colin Ray Anderson, Janneke Bruil, M. Jahi Chappell, Csilla Kiss, Michel Patrick Pimbert

**Affiliations:** 6 Centre for Agroecology, Water and Resilience, Coventry University, Wolston, UK; 7 Cultivate! Collective, Bennekom, The Netherlands; 8 Centre for Agroecology, Water and Resilience, Coventry University, Coventry, UK

**Keywords:** Nested markets, Traditional markets, Corporate power, Global food system, Subsistence

## Abstract

In this chapter we examine the importance of systems of economic exchange for agroecology. These include the practices and processes by which agricultural products move from producers to various users and by which agri-food producers acquire inputs that cannot be produced on the farm. We review the importance of traditional systems of exchange (such as informal markets and barter systems), subsistence (or family and community self-provisioning) and ‘nested markets’ that are embedded in democratic social relations for agroecology. These markets thicken networks of solidarity and relations of reciprocity in territories. Nested markets value the ecological, social, economic and political functions and outputs of agroecology and support the development of trust-based networks. Regrettably, mainstream food markets favour large volumes and standardization and exclude most agroecological producers.

We use the term *systems of economic exchange* (or in shorthand: *systems of exchange*) in food and farming to mean the practices and processes by which agricultural products move from producers to various users and by which agri-food producers acquire inputs that cannot be produced on the farm. Systems of exchange are thus “the rules-based exchanges of value in specific contexts where the rules can come from public regulations, private contracts, civic norms or cultural customs” (FAO 2016). They include both formal market mechanisms and informal exchange between agricultural producers of seeds, livestock breeds, labour and more. The extent to which these systems of exchange are accessible, fair, profitable and fulfilling for food producers helps to determine the quality of agroecological transformations.

Agroecology is not anti-trade or against markets *per se*. To be viable, however, it requires systems of exchange that differ starkly from the capitalist, corporate-led systems of exchange that pervade the dominant regime. The existence of appropriate and robust systems of exchange, including different types of markets, state provisioning, barter, gifts and self-sufficiency, are all important enablers of agroecology. Longstanding traditional systems of exchange and the creative construction of newer ‘alternative food systems’, relations and markets represent a key opportunity for agroecological transformations.

## Enabling Conditions

Agroecological production is based on the integration of a diversity of crops and of livestock; it thus relies on forms of economic exchange compatible with small volumes of many different farm products and local diets. By sustaining a diversity of domesticated and wild foods, agroecological practices themselves are an important enabler of systems of exchange at scales from farm plots to the wider landscape and the commons. Farmers’ agroecological practices enhance available dietary diversity by creating micro-environments for growing many different crops and livestock on farms and neighbouring landscapes as well as on the commons—grasslands, forests, wetlands. In addition, these practices sustain key ecological functions at different spatial scales, such as pollination, natural pest control, waste decomposition, water filtration and carbon sequestration (IPBES 2019). These so-called environmental goods and services sustain the material basis of systems of economic exchange important for food and livelihood security.

To support systems of exchange that advance agroecological transformations, it is important to value and build on existing community networks and cultures. Traditional systems of exchange (such as informal markets and barter systems) that have evolved within traditional communities, ecosystems and culture are, although undervalued, a good basis for enabling systems of exchange for agroecology. For example, wild resources found on farms and common lands are often incorporated into agroecological systems. Wild edible plants and animals are particularly important to indigenous people’s food and livelihood security as well as that of the rural poor, women and children, especially in times of stress such as drought, shifts in land and water availability or ecological change. With much less access to land, capital and labour, these groups rely on systems of exchange involving wild diversity.

Also key for agroecology are new markets, networks and economic processes that are embedded, or ‘nested’, in local territories and social relations, for example around definitions of food quality that are mutually agreed by producers and consumers (Jan Douwe van der Ploeg et al. 2012). The Beijing County Fair in China is one example of such new nested markets (Box 6.1). Most commonly, nested markets remove intermediaries as much as possible and are oriented towards direct connections between producers and consumers that build mutual understanding and new solidarities. The Food and Agriculture Organization of the United Nations (FAO) (2018) found that in nested markets, actors are “recapturing value through direct contact, but also through a diversification of their market channels”. Nested markets recognize and promote the multiple benefits of agroecological food production—biodiversity, human and ecological health and natural resource management, for instance—which are otherwise undervalued. They also accommodate the diversity of outputs generally produced in agroecological systems, allow for local self-determination and meet the material needs of food producers. This often makes nested markets more attractive for agroecological food producers than conventional markets and global value chains.

 Nested markets exist in many forms and under many names. For example, ‘alternative food networks’ broadly include newly emerging networks of, and relations between, producers, consumers and other actors that embody alternatives to the more standardized industrial systems of food exchange (Kneafsey and Holloway 2008). Some examples of nested market arrangements include participatory guarantee systems, restaurants purchasing food directly from farms, vegetable boxes, farm shops, self-harvest fields and public food procurement (e.g. in university, government and hospital cafeterias). Community-supported agriculture (CSA) is another such arrangement currently on the rise. The international CSA network Urgenci, with members on every continent, defines CSA as “local solidarity-based partnerships between producers and consumers” centred on trust and shared risk.

Building nested markets for agroecology is a case of step-by-step processes based on local resources, in which additional assistance from the state may play a strategic role (Jan Douwe van der Ploeg et al. 2012). Crucial steps in constructing agroecological markets include the diversification of relations and channels (such as through new partnerships with restaurants, educational establishments and consumer groups), resolving post-harvest conservation and storage problems, developing innovative small-scale processing of traditional varieties and carrying out active promotion of these initiatives. The latter often happens by strategically positioning products and creating awareness among consumers, mainly through media, personal communication, farm visits, local events and education. Nested markets thus can have a positive impact on social cohesion, the economic vitality of territories and carbon footprints. They “counter distance with proximity, artifice with freshness, anonymity with identity and genuineness, standardization with diversity and inequality with fairness” (Jan Douwe van der Ploeg et al. 2012).

However, nested markets in some cases replicate the extractive, competitive and exclusionary dynamics and relations of the dominant food system. Based on a heterodox view of economics, the framework for these markets argues that they “coexist with other (conventional) markets and struggle with these for space and legitimacy”, and “constitute concrete spaces of interaction between specific actors, which are constructed and reproduced within the conventional markets, that is, within the capitalist mode of production” (Sonnino and Marsden 2006). The politics in some farmers’ markets and CSAs, for instance, have been found to be driven as much by profit-seeking and individualism as by logics of solidarity and trust (Hinrichs 2000) or to echo the exclusionary dynamics underlying racial capitalism (Slocum 2007).

 Nested markets are vital, but it is important to view them critically and to question their political underpinnings so that they can more effectively foster agroecological transformations. Some forms of such markets are more explicitly opposed to capitalist and extractive economies, for example solidarity economics, de-growth, and eco-feminist, indigenous and anarchist economics.

In agroecology, not only products but also cultural traditions, ideas, visions and knowledge are exchanged. As Stephen Sherwood et al. (2018, p. 5) note, an agroecological market is “a site of social creativity where people situate and territorialize their abilities to affect and be affected”, allowing them to shape their own socio-material conditions. The authors illustrate this through a case study of the Carcelen Agroecology and Solidarity Fair in Quito. While state institutions tried to enforce official norms and standards around production, hygiene and price, participants in the fair were “renewing a sense of self and collectivity”. Through this they generated relationships focused not only on a need for calories and food security but also on new values connected to cultural expression, health, environmental sustainability and a sense of community. This is one of many ways in which an agroecological approach may reveal the first stirrings of new “regimes”—“food from somewhere” as opposed to corporate “food from nowhere” (McMichael 2009).

 Labelling has been promoted as another mechanism for upscaling and securing markets for sustainable food. While third-party labels and certificates have indeed provided important support for the scaling up of different approaches to sustainability in agriculture, such as organic agriculture and fair trade, the mechanism is contested. For producers who want to participate in certification schemes, problems often arise in relation to cost or demands to conform to externally agreed standards that may have little to do with agroecology. If people are urged to trust a label rather than engage, discern and participate in building local food systems, it can reduce citizens to passive consumers and effectively decouple place from production. So, while labelling may have some role to play in enabling systems of exchange for agroecology, a critical question remains: *who* is responsible for developing, implementing and controlling standards and evaluating which are necessary?

Alternatives to third-party labels exist. To ensure a certain level of food safety and quality while not losing control over their production system, producers in countries like China, France, India and Italy have come together to collectively agree on production methods and standards. These autonomous mechanisms are called participatory guarantee systems (PGSs): locally focused quality assurance systems in which producers self-certify, in some cases in collaboration with consumers (for an example, see Box 6.1).

### Box 6.1 The Beijing County Fair—Building a Commitment to Sustainable Food

**Fig. 6.1:**
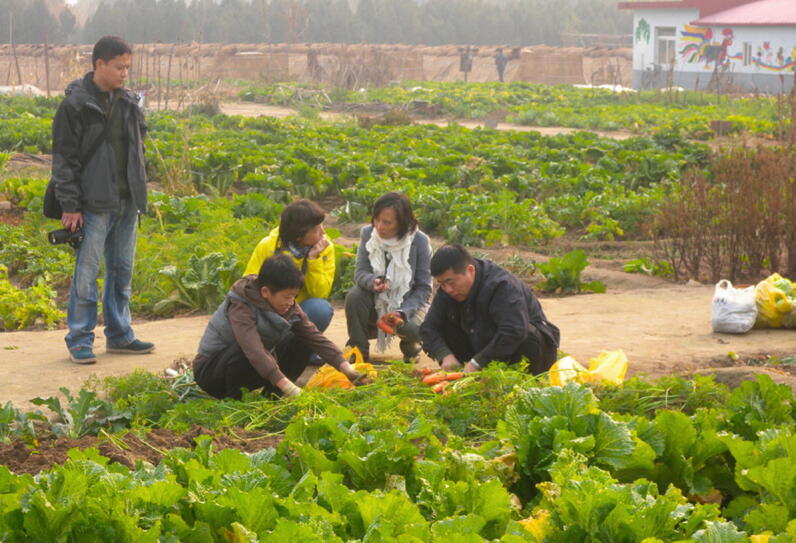
CSA members of Little Donkey farm (Beijing, China) harvesting carrots (*Photo credit*: Jan Douwe van der Ploeg)

The Beijing County Fair in China is an example of a nested market that supports agroecology transitions. It was first organized by local consumers and artists in 2010, and by 2015 it was run by 11 full-time staff. Within a decade, it has developed into the most active and influential ecological farmers’ market in China. One of its managers notes that in 2017 her team organized 154 markets, each time involving about 20 small- to medium-scale ecological farms and 10 smallholder food processors. Other than farmers’ markets, the same team also runs 2 grocery stores and an online shop, selling the produce of over 70 farms and 20 food processors’ facilities.

The Fair has rebuilt trust between individual food producers and consumers and has developed the trust of consumers in institutions (Wang et al. 2015). One of the Fair’s key tools in this context is a PGS. In 2014, the Fair started to experiment with a PGS by developing a farm information form completed by about 30 farms and checked during farm visits, as well as regulating and increasing the frequency of farm visits (Jiang 2015). The form is used to holistically evaluate a farm in terms of technical practice and social aspects such as ownership structure, employment and marketing approaches. Transparency is key to participation in the Fair: the forms are displayed at the farmers’ market and also available online.

The Fair is the PGS pioneer in China, but not the only body to adopt it. At the start of 2018, 18 farmers markets (including the Fair), social enterprises and buyer groups across China established a PGS network called ‘Clover’ to enable collective learning on standards, joint farm visits and communication activities.

*Source:* Xu Ye and Mindi Schneider, the International Institute of Social Studies (ISS), The Hague, Netherlands

PGSs are the most widely recognized alternative food certification systems. They are built on a foundation of trust, social networks, knowledge exchange and local control. They keep the costs of certification low. They also respond to the need for clarity on what ‘agroecological’ means, bringing agroecological actors together in territories to negotiate its meaning as it applies to particular contexts. PGSs can also challenge the assumptions of the dominant regime that underlie third-party certification, such as the prioritization of export-oriented production and the idea that only formally trained experts can make valid assessments of quality. As local institutions for collective decision-making, PGSs can therefore be considered a tool for strengthening innovation in agroecology, for challenging the dominant regime in food and farming and for moving to a commons model and away from commodification of agriculture and its products (Vivero-Pol et al. 2018).

When food safety regulations and validation processes are tailored to small-scale agricultural producers using an agroecological approach, agroecological systems of exchange inevitably benefit. Conversely, rigid and uniform rules on, for example, food safety and plant disease control can severely limit the circulation of artisanal products of small-scale producers and often fail to improve food safety (McMahon 2013). To ensure consistent quality in organic food, legislation and governmental standards have been established for production, processing, trading, monitoring and certification—for example, the European Council Regulation on Organic Farming No. 202, Brazil’s organic farming legislation of 2003 and Japan’s Agricultural Standards for Organic Agricultural Products and Their Processed Foods.

Some of these regulations, however, were constructed for large-scale farming and processing and could undermine the specific production model of small-scale agroecological producers. For example, organic food production rules and certification rarely take into account the proximity of production and consumption as a safety feature for the nutritional quality of foods (Vogl et al. 2005). For agroecology to thrive, regulatory mechanisms for food safety and quality must allow for regional definitions while supporting small-scale producers’ knowledge and socio-technical experiments in sustainability and resilience.

Many actors around the world have called for regulatory, financial and infrastructure state support for markets for agroecology. Indeed, governments can play enabling roles. In light of the broadly recognized human right to food, food cannot be conceived as a commodity like any other. It is thus essential that states intervene in markets. For example, state support was essential for the development of four different types of markets for agroecology in China: the export-oriented market for organic produce; the domestic market for certified food; the localized market for traditional agriculture and typical regional products; and markets for agro-tourism. While many of these markets started off as experiments by farmers, after learning and adjustment they were integrated into government programmes. Each now plays a distinct role in supporting agroecology (Ye et al. 2010).

There are various forms of government support for systems of exchange. One is through public food-procurement programmes such as the Program for Food Acquisition from Family Farming (PAA) in Brazil (Box 6.2). Or states can lend financial, logistical or promotional support to markets for agroecology and thus increase their visibility and viability. A key role for governments here is establishing infrastructure that overcomes impediments in transportation and information networks, for example by building cold-storage systems for fresh fruits and vegetables.

### Box 6.2 Public Food Procurement as a Motor for Agroecology in Brazil

 Brazil’s Program for Food Acquisition from Family Farming (PAA) was established in 2003 as part of former president Luiz Inacio Lula da Silva’s Zero Hunger Strategy. It has a dual objective: to bring quality food to the socially most vulnerable sectors of society *and* to strengthen family farmers, even the most impoverished. Notably, the PAA has stimulated crop diversification and helped to open new marketing channels. With the same budget, it has also had positive impacts in other sectors, such as biodiversity conservation, public health and addressing climate change. For Brazilian social movements, the PAA has been the most innovative and effective public policy for agroecology.

Moreover, since the 1940s Brazil has been running the National School Feeding Program (PNAE), explicitly aimed at creating an institutional market for Brazilian agricultural producers. Since 2009, the PNAE requires that 30% of purchases come from local family farmers, offering a price premium for agroecologically produced food.

The PAA followed an upward path for over a decade. By 2016, it had reached sales of R$850 million (approximately 150 million euros) buying and distributing more than 297,000 tons of food from 380 different products in all the Brazilian states, and benefiting approximately 185,000 farmers’ families. This was possible because the PAA involved more than 24,000 social organizations that worked to help families in situations of social vulnerability. In that same year, however, brutal budget cuts began.

Now, in 2020, the PAA budget is reduced to less than R$100 million. The procedures have become very bureaucratic, making participation of the poorest farming families extremely difficult (Oldekop et al. 2015). Social movements are currently, in the midst of the COVID-19 pandemic, organizing for PAA to be revitalized and the PNAE to be improved. Their goal is to resume the original modalities of the PAA programme and increase the budget to R$1 billion by the end of 2020.

*Source:* Prepared in collaboration with Paulo Petersen, AS-PTA, Brazil

So, the role of governments in promoting markets for agroecology can be key. However, when markets are constructed in a non-participatory manner, they may become counterproductive, as barriers to inclusion, bureaucracy, paperwork and costs may emerge. Moreover, care must be taken that these markets continue to support diversified agroecological food production, especially when the market seems to be shifting to larger volumes or towards export. Similarly, in terms of nutrition and food security, policies to enhance agroecology for sustainable food systems must promote production for household consumption over that for commercial interests.

In addition to the ‘downstream’ side of systems of exchange (i.e. moving goods from producers to users and consumers), agroecology also demands appropriate upstream systems of exchange. The majority of external, capital-intensive inputs need to be gradually displaced by knowledge-intensive practices based on natural processes such as on-farm production of organic fertilizers, the use of natural processes for pest control, intercropping and soil management. These have reduced farmers’ dependence on a host of industrial-chemical inputs and their levels of debt. In one example, savings from lower expenses on farm inputs allowed 386 out of 487 households surveyed in Andhra Pradesh, India, to reclaim their mortgaged farmland (Gregory et al. 2017).

There may still be inputs that farmers cannot derive on the farm but need to acquire from other producers through dynamic exchange of seeds, breeding stock, feed, labour, nutrients and tools. These systems of exchange may consist of formal market-based mechanisms or informal relations. Community seed collecting, practised in regions from Asia to Africa, is one such informal system, involving the exchange and systematic sharing of seeds as well as arrangements to exchange manure and feed. Such initiatives are enabled in contexts where civil society networks are developing open source seed systems (Montenegro de Wit 2017), where there is an active movement to reject biopiracy and genetically modified seeds and where peasant seed networks already exist and are being defended (Peschard and Randeria 2020). These points drive home yet again how important power and politics are in the development of agroecological networks.

Another inspiring example is rooted in the idea that farmers themselves are innovators. In the network of L’Atelier Paysan in France, farmers collaborate with engineers, IT specialists and mechanics to develop and exchange tools and self-built machinery for agroecology-based farming. Through the sharing of farm-based inventions, the initiative makes agroecology transdisciplinary. L’Atelier Paysan also engages in farmer-driven projects to build or renovate agricultural buildings. The network’s designs for new farm tools and machinery are all disseminated as open source materials, and it runs courses and produces educational materials to share skills and ideas. In these ways, L’Atelier Paysan builds an upstream system of exchange that affirms the principle of technological sovereignty within and between territories.

## Disabling Conditions

One of the most significant barriers to developing agroecology is the absence, or erosion, of appropriate systems of exchange, coupled with the growth of specialized, export-oriented value chains. These mainstream food markets generally demand large volumes of product and standardization, reinforced by policies that emphasize economies of scale, strategic export commodities and integration into global value chains, which many agroecological producers cannot, or opt not to, engage in (IPES-Food 2016; van der Ploeg 2018).

There are many reasons why they don’t. Because agroecological approaches focus on crop and genetic diversity, farmers using the system may only rarely produce sufficient quantities of uniformity in single crops to solely participate in export markets and global value chains. Further, commodity prices are often at or below the cost of production. This provides clear benefits to agribusinesses in processing and retail, for instance, but it traps small-scale farmers in cycles where they must “go big or get out”—specialize or be excluded from export markets (Howard 2016). In addition, the current drive to harmonize food safety standards across the world often favours multinational capital and marginalize local small-scale producers, yet creates systemic “un-safety, poor health and a future of food insecurity for many” (McMahon 2013).

Thus, globalized market arrangements do not work well for agroecology. The prices do not reflect the costs, and important non-market values central to agroecological principles are driven out—equity, shared social welfare, solidarity, kinship, reciprocity, culture and traditions among them. An example, described by Alexander Day and Mindi Schneider (2017), shows how the contemporary political economic context in China, which pushes intensified modernization, has compelled agroecological networks to follow the same market logic as state policy-makers—specifically to “focus on niche marketing to the urban middle class, without seeking to transform rural social relations” (Day and Schneider, 2017 p. 1223). These lock-ins pose challenges to markets for agroecology, such as an inability to respond to rising demand because of inconsistent levels of agroecological production, lack of adequate logistics for distribution, low consumer consciousness, limited public sector support and unfair price competition (FAO 2018).

Against all this stands the fact that a minority of the world’s food is directly exchanged in global markets: only 12–17% of the total volume crosses an international border between production and consumption (Chappell 2018, p. 204n8). Many states and policies, driven by concerns about food security, attempt to change this and explicitly prioritize the integration of small-scale food producers into global markets rather than encourage the development of diverse local markets.

But such efforts to make global value chains more ‘inclusive’ tend to benefit only a small number of farmers worldwide—10% at most—who tend to be well off, educated, strongly oriented towards commercial agriculture and living close to urban areas and infrastructure (Seville et al. 2011). On the consumer side, international trade has mainly benefited wealthy consumers in high-income countries while marginalizing communities in low-income countries who continue to be unable to afford the diversity available on global markets. In Bangladesh, the commercialization of agriculture and the continued forced integration of farmers in the market economy regime are considered to be at least partly responsible for today’s high rates of malnutrition among rural people (Misra 2017).

The global overproduction of food and concomitant decline in prices typically harm farmers’ livelihoods. Farmers will usually increase production to make up for lower prices for each unit they produce (Chappell 2018, pp. 42–44). In practice, this means that producers are often reluctant or unable to get off this ‘treadmill’ and may be deterred from shifting to agroecological practice. But it is immensely profitable for corporations, as they are able to sell ever more inputs and buy ever-cheaper agricultural products (Chappell 2018). This in turn helps to lock-in the current regime and block transition, as farmers are often encouraged to adopt new technologies in order to boost production. Another problem with global overproduction is that it forces producers to raise crops or livestock months before they know what the selling price will be.

 Markets that provide inputs for agriculture, aided by schemes subsidizing external inputs, pose hurdles for agroecological transition. The concentration and consolidation of these markets has been called “one of the most pressing concerns” related to agricultural industrialization (Hendrickson et al. 2017). Here, again, large corporations make significant profits while pushing farmers into growing resource-intensive, environmentally destructive monocultures for very low prices, often below production cost. The cost of external inputs is a major burden for producers, who turn to subsidy schemes; they then often accelerate and increase their use of fertilizers, pesticides, commercial seeds, non-locally adapted livestock genetics and imported feed. Paying for inputs reduces profit margins, which may trigger a need for credit and risk insurance. (This also happens with livestock production that is dependent on costly external inputs such as feed, medicine or capital-intensive installations such as stables.) As with overproduction and its impact on farmers, a cycle of debt, consolidation and industrialization can result (Chappell 2018; Howard 2016).

To enable farmers to access external inputs, many countries have established public subsidy programmes. A 2016 study by the African Centre for Biodiversity on the effects of state-led farm input subsidy programmes in ten countries in southern Africa found these to be largely ineffective, as a result of grabbing by elites and diversion, for example through theft or sale by beneficiaries (Africa Centre for Biodiversity 2016). According to the study, the subsidies’ direct contributions to higher yields and reduced food prices failed to directly benefit the poor and most vulnerable, who are mostly women. Importantly, the input subsidy programmes increase rural communities’ dependency on external inputs, impeding any move to agroecology.

Removing such government subsidies for agro-industrial inputs can eliminate perverse incentives that keep farmers hooked on agro-industrial networks. For example, a programme launched in 2003 by the government of Sikkim state in India reduced subsidies for agrochemicals by 10% each year. By 2007–2008, they were eliminated, and by 2009, the sale of all agrochemical products was phased out (Gregory et al. 2017). In concert, the state aimed to support the development of a bio-input industry and to develop markets for the organic products of Sikkimese agriculture; however, unfortunately many of these policies were ill-conceived and in practice served to undermine agroecology (Meek and Anderson 2020; see Box 10.1 in Chap. 10.1007/978-3-030-61315-0_10).
